# [Corrigendum] High expression of RELM-α correlates with poor prognosis and promotes angiogenesis in gastric cancer

**DOI:** 10.3892/or.2025.8894

**Published:** 2025-04-02

**Authors:** Ping Chen, Deshou Zhao, Weiyi Wang, Yongping Zhang, Yaozong Yuan, Lifu Wang, Yunlin Wu

Oncol Rep 34: 77–86, 2015; DOI: 10.3892/or.2015.3943

Subsequently to the publication of the above paper, and a corrigendum (doi.org/10.3892/or.2018.6933) that was published in 2018 to account for duplicated data panels in Fig. 6, an interested reader has drawn the authors' attention to the fact that the β-actin bands featured in Fig. 3B of the above paper were strikingly similar to control data which appeared in Fig. 2D of a paper that had already been published in the journal *Oncotarget*, which featured one author in common.

After having re-examined their original data files, the authors realized that the β-actin bands in Fig. 3B of the above paper had been inadvertently assembled incorrectly. The revised version of Fig. 3, now featuring all the correct data for Fig. 3B, including an adjusted version of the histograms in this figure part after having re-quantified the data, is shown on the next page. Note that the corrections made to this figure do not affect the overall conclusions reported in the paper. The authors are grateful to the Editor of *Oncology Reports* for allowing them the opportunity to publish this further Corrigendum, and apologize to the readership for any inconvenience caused.

## Figures and Tables

**Figure 3.**
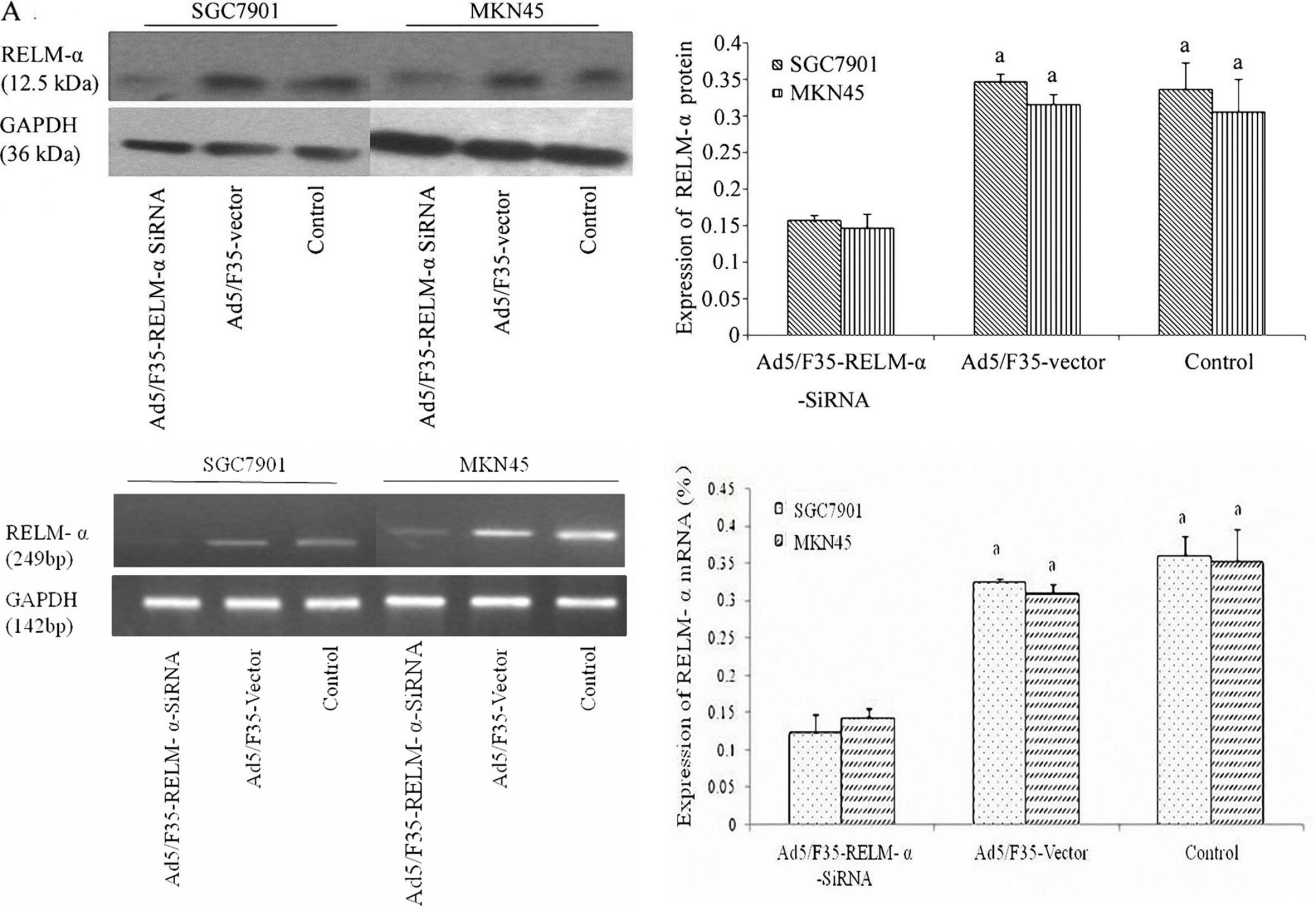
RELM-α expression was determined in gastric cancer cell lines SGC7901 and MKN45 in each treatment group. (A) Western blot analysis demonstrated that RELM-α expression was downregulated in the cells after infection with Ad5/F35-RELM-α-siRNA at 24 h. The control cells and the cells infected with Ad5/F35-vector showed high expression of RELM-α at 24 h. (B) mRNA expression of RELM-α showed changes similar to the results of the western blot analysis. ^a^P<0.01 vs. the Ad5/F35-RELM-α-siRNA-treated group. RELM-α, resistin-like molecule-α.

